# Case Report: Safe and Effective Sublingual Birch Allergen Immunotherapy in Two HIV-Positive Patients

**DOI:** 10.3389/fimmu.2021.599955

**Published:** 2021-07-27

**Authors:** Elena Latysheva, Evgeniya Nazarova, Tatiana Latysheva, Natalia Ilina

**Affiliations:** Institute of Immunology, National Research Center Institute of Immunology Federal Biomedical Agency, Moscow, Russia

**Keywords:** allergen immunotherapy, HIV positive, birch allergen, case report, sublingual

## Abstract

Allergen-specific immunotherapy (AIT) is a safe, effective treatment for respiratory allergies (such as moderate-to-severe allergic rhinoconjunctivitis) that are not controlled by symptomatic medications. The indications and contraindications for AIT have been defined in international guidelines and consensus statements. However, some of these contraindications are not evidenced- based but have been deduced from the theoretical risk of an interaction between AIT disease-modifying effect and immune or inflammatory comorbidities. In the absence of clinical trial evidence, the accumulation of experience as case reports can narrow the spectrum of absolute contraindications. The majority of international guidelines list HIV infection as a contraindication to AIT. Here, we describe two cases of safe, effective sublingual birch pollen AIT in HIV-positive patients undergoing concomitant antiretroviral therapy. A 32-year-old female and a 63-year-old male sensitized to tree pollen and with clinically confirmed birch pollen allergy underwent pre- and co-seasonal sublingual birch pollen AIT for three and two pollen seasons, respectively. The therapy was associated with a marked reduction in the frequency and intensity of allergic symptoms, and the reduced use of (symptomatic) rescue medication. Mild, local, treatment-emergent adverse events were noted throughout the course of treatment but resolved spontaneously. No serious adverse events were reported. In particular, there were no obvious harmful effects on the patients’ immune status or viral load. Hence, sublingual birch pollen AIT proved to be effective and safe in two HIV-positive patients.

## Introduction

Allergen-specific immunotherapy (AIT) is the key method for the treatment of IgE-mediated respiratory allergies (1–4). The indications for this type of treatment are clearly defined, do not significantly change over time, and do not differ greatly from one country to another – in contrast to the contraindications (5). Firstly, the list of contraindications includes comorbidities/concomitant treatments that may contribute to the development of adverse reactions associated with AIT or reduce the efficacy of AIT treatment. Secondly, the list takes account of the potential risk of aggravation of a comorbidity by AIT. In recent years, the number of contraindications has steadily decreased as more safety data are collected and with the emergence of AIT formulations that minimize the risk of anaphylaxis (6). The majority of absolute contraindications have become relative, and many of them are transient. Nevertheless, there is a lack of data from large placebo-controlled studies of the efficacy/safety of AIT in patients with certain concomitant diseases. Hence, the physician’s decision to initiate AIT is based on (i) his/her personal experience and (ii) case reports in the literature.

Although immunodeficiencies (both primary and secondary), are listed as contraindications in most position statements, there are no objective data on the possible effects of AIT in these settings (2, 7, 8). The use of AIT in HIV-positive patients has been restricted because of the immunotherapy’s potential effects on the activation of infected CD4+ cells. To date, only a few descriptions of clinical cases and one cohort study of 13 patients with a control group are available in the literature. However, the available data indicate that there is no evidence of adverse effect of AIT on the course of HIV infection (viral load and CD4+ count), while the effectiveness of therapy was noted by all authors (9–11). However, the currently available data suggest that this risk is low when the patient is receiving highly active antiretroviral therapy (HART). Nevertheless, there are few publications about AIT in HIV-positive patients, and the lack of data prevents one from drawing statistically robust conclusions about the efficacy and safety of this treatment method. Furthermore, the published studies focused on safety (rather than efficacy) and had a short follow-up period.

Here, we report on birch pollen sublingual AIT in two HART-experienced, HIV-positive, patients. The length of pre- and co-seasonal treatment was 3 and 2 years, respectively. The first patient was followed up for 3 years after the end of the course of AIT.

## Case Presentation #1

A 32-year-old female complained of symptoms of severe rhinoconjunctivitis, cough, and episodic shortness of breath in the spring, together with year-round oral pruritus following the consumption of drupes, nuts, and pears. The patient also reported symptoms of rhinoconjunctivitis following exposure to large amounts of dust. Symptoms of rhinoconjunctivitis in spring appeared from the age of 22 and had a pronounced increase (including the episodes of shortness of breath) during the last 5 years after changing the place of residence. The patient’s ongoing treatment with symptomatic medications (antihistamines, topical nasal and inhaled glucocorticoids (GCs), and (for exacerbations) oral GCs) was only partially effective. Allergen-specific IgE assays (Immunocap) revealed sensitization to birch pollen and house dust mite allergens. The patient was diagnosed with mild bronchial asthma and severe intermittent allergic rhinoconjunctivitis. Although the patient had consulted a number of allergists, AIT had always been refused due to the HIV infection and the lack of efficacy/safety data for AIT in HIV-positive patients. At the time when the patient first consulted in our establishment (the NRC Institute of Immunology FMBA, Moscow, Russian Federation), she had been receiving active HART for 8 years. A general clinical examination did not identify any significant abnormalities. The allergic examination revealed an increase in total IgE to 267 IU/ml, specific IgE to birch pollen 49.6 IU/ml (class 4), respiratory function was normal (FEV1>70%). A PCR blood test showed that the HIV viral load was below the limit of detection. The patient’s CD4+ cell count was normal (**Table 1**).

**Table 1:** Development of CD4+ Cell Count, HIV RNA, questionary data.

Patient 1	Before SLIT	1st year of SLIT	2nd year of SLIT	3rd year of SLIT	Follow up 1st year	Follow up 2nd year	Follow up 3rd year
Scale for assessing the mean adjusted symptom score with medication intake	26	16	6	6	2	4	2
ACT	22	25	25	25	25	25	25
VAS	10	7	2	3	1	1	1
CD4+/μl	932	916	935	980	976	991	985
Viral load	negative	negative	negative	negative	negative	negative	negative

After the patient had given her written, informed consent, we initiate birch pollen sublingual AIT. House dust mite AIT was not indicated because the symptoms were mild and infrequent. The patient received three consecutive courses of pre-co-seasonal birch pollen sublingual AIT [Staloral^®^ Birch from Stallergenes (Antony, France); daily maintenance dose: 300 index of reactivity (IR)] from December to May, followed by 3 years of follow-up. The viral load and CD4+ cell count were monitored at 3-month intervals during the first year of treatment and at 6-month intervals during the subsequent years of treatment and follow-up. No positive PCR results and no negative trends in the CD4+ cell count were observed at any time during treatment and follow-up. During each tree pollen season, the patient kept a symptom diary and also noted any adverse events and concomitant medications. During the first course of treatment with birch pollen sublingual AIT, the patient reported moderately severe but transient local adverse events (sublingual pruritus) that either self-resolved or resolved following treatment with an oral antihistamine. No systemic reactions were reported. During the two following seasons of AIT, the patient did not report any adverse reactions and did not require antihistamines.

Positive effects on allergic respiratory symptoms were seen after the first course of AIT and maintained the entire observation period: the patient noted a reduction in the severity and duration of rhinoconjunctivitis symptoms and did not experience any shortness of breath or cough during spring at any time during treatment or follow-up. During the entire follow-up period the patient never used beta2-agonists or inhaled maintenance therapy. A fourth course of AIT was planned but not initiated because the patient had become pregnant. Given that the seasonal manifestations of allergic rhinoconjunctivitis and asthma were fully controlled after the third year of pre- and co-seasonal AIT, we decided to discontinue the treatment but continue the monitoring. During the three subsequent birch pollen seasons, the patient merely noted the occasional use of antihistamines and no other symptomatic medications.

## Case Presentation #2

A 63-year-old male complained of rhinoconjunctivitis symptoms in the spring for the previous 10 years, together with pruritus of the roof of the mouth and the ears following the consumption of drupes, nuts, apples, and carrots. Treatment of these symptoms with oral antihistamines, nasal GCs, and antihistamine eye drops was only partially effective. The patient was reportedly sensitized to tree, weed and grass pollen, but had no symptoms during weed and grass pollination season. The patient was HIV-positive and had been refused AIT for many years. When the patient consulted in our institution, he underwent a comprehensive allergologic and immunologic work-up. Total IgE was 375 IU/ml. The Immunocap assay confirmed sensitization to tree 74.9 IU/ml (5 class), weed 18.6 IU/ml (4 class) and grass pollen 14.3 IU/ml (3 class). The patient was diagnosed with severe intermittent allergic rhinoconjunctivitis, he had been receiving active HART for 8 years, and the disease progression had been monitored by an infectious disease specialist.

In view of the incomplete symptom control with pharmacologic therapy and the absence of signs of viral replication or clinically significant changes in lymphocyte subsets (i.e. a CD4+ count within the normal range), we recommended birch pollen sublingual AIT. After the patient had given his written, informed consent, he underwent two courses of pre- and co-seasonal therapy Staloral^®^ Birch from Stallergenes (Antony, France); daily maintenance dose: 300 IR) from December to May. CD4+ cell counts and PCR tests for the HIV load were performed at 3-month intervals during the first year of treatment, and then at 6-month intervals during the second year. As in case #1, no positive PCR results and no negative trends in the CD4+ cell count were observed at any time during treatment and follow-up.

During the treatment period, the patient experienced the local adverse reactions commonly associated with sublingual AIT (pronounced pruritus and moderate edema in the sublingual region starting 1 or 2 minutes after administration and persisting for 25 minutes). However, the symptoms were managed with antihistamines and did not modify require modification of the AIT regimen. No systemic reactions were reported.

Based on the patient diary, an improvement was noted following the first course of pre- and co-seasonal AIT: mild rhinoconjunctivitis symptoms were successfully managed with standard doses of oral antihistamine, and the patient did not require topical GCs or topical antihistamines. Following the second course of AIT, the patient noted a further reduction in the severity and duration of rhinoconjunctivitis symptoms, which were fully controlled with oral antihistamines. The patient was advised to continue AIT, to monitor the viral load and CD4+ cell count, and to keep a diary of symptoms and concomitant medications (**Table 2**).

**Table 2:** Development of CD4+ Cell Count, HIV RNA, questionary data.

Patient 2	Before SLIT	1st year of SLIT	2nd year of SLIT	Follow up year
Scale for assessing the mean adjusted symptom score with medication intake	25	12	3	4
VAS	9	5	1	1
CD4+/μl	519	623	631	674
Viral load	negative	negative	negative	negative

## Discussion

The emergence of effective ART has enabled HIV-positive patients to enjoy much the same quality of life and life expectancy as the general population (12). However, allergies are not rare in people living with HIV (13–15). The lack of data on the efficacy and safety of AIT in HIV-positive people patients means that these patients are typically refused access to an effective, disease-modifying therapy. The putative negative impact of AIT on HIV infection is purely theoretical; there are no data from controlled trials, and very few case reports. Although the majority of international guidelines and position statements on AIT consider HIV infection to be a relative contraindication only, some (including those adopted in Russia) list immunodeficiency as an absolute contraindication (16).

In general, physicians are not ready to take responsibility for this decision, as demonstrated by the cases described above. Over many years, our two patients were refused AIT by various different medical institutions and were advised to use symptomatic drugs – drugs that did not control their symptoms. According to Larenas-Linnemann et al., one in two physicians considers HIV infection to be an absolute contraindication to AIT - even in countries where HIV infection is officially only a relative contraindication (17). A survey of 1085 AIT-prescribing members of the American Academy of Allergy, Asthma & Immunology showed 48% considered HIV infection to be an absolute contraindication to AIT.

Despite the lack of data on the safety and effectiveness of AIT in patients with HIV infection, the available publications indicate the benefit of this type of the therapy. In their study, E Lemoli et al. compared 13 patients with HIV who received sublingual immunotherapy (SLIT) tablet (Oralair, Stallergenes^©^) with patients who received only symptomatic treatment. Clinical efficacy data showed a significant improvement in patients treated with SLIT compared to the control group in total combined score (TCS), the sum of symptoms-drug score and the quality of life questionnaire (QoL). No negative effect on the course of HIV infection was noted (11).

All case studies of HIV patients treated with AIT describe patients already under antiretroviral therapy before starting AIT. To date, there is no data on whether AIT will be as safe in patients without ART. However, taking into account that any concomitant pathology in decompensation is an absolute contraindication, this issue is irrelevant.

Our data confirm that in ART-experienced patients with no signs of viral replication and with a normal CD4+ cell count, AIT was not associated with aggravation of HIV infection, an elevated risk of adverse reactions, or poor efficacy. In the two present cases, our long-term follow-up after pre- and co-seasonal birch pollen sublingual AIT evidenced lasting symptom relief (for 3 years of treatment and 3 years of follow-up in case #1, and for 2 years of treatment in case #2).

The analysis of the presented clinical cases showed that the side effects in HIV patients receiving ART do not differ from those observed in the general population and correspond to those described in the instructions for the drug.

The limitation of the article is the review of only 2 clinical cases, which do not allow us to draw reliable conclusions, but considering the small number of publications on AIT in people living with HIV, we believe that our clinical experience may help other physicians to decide whether to initiate AIT in an HIV-positive patient.

## Data Availability Statement

The raw data supporting the conclusions of this article will be made available by the authors, without undue reservation.

## Ethics Statement

Written informed consent was obtained from the individuals for the publication of any potentially identifiable images or data included in this article.

## Author Contributions

EL - examination and treatment of patients and writing an article. EN - examination and treatment of patients and article review. TL - control of the treatment process and article review. NI - control of the treatment process and article review. All authors contributed to the article and approved the submitted version.

## Funding

The authors declare that this study received funding from Stallergenes Greer. The funder was not involved in the study design, collection, analysis, interpretation of data, the writing of this article or the decision to submit it for publication.

## Conflict of Interest

The authors declare that the research was conducted in the absence of any commercial or financial relationships that could be construed as a potential conflict of interest.

## Publisher’s Note

All claims expressed in this article are solely those of the authors and do not necessarily represent those of their affiliated organizations, or those of the publisher, the editors and the reviewers. Any product that may be evaluated in this article, or claim that may be made by its manufacturer, is not guaranteed or endorsed by the publisher.
