# Supplementary material for: Case Report: Safe and Effective Sublingual Birch Allergen Immunotherapy in Two HIV-Positive Patients
Source: Front Immunol. 2021 Jul 27;12:599955. doi: 10.3389/fimmu.2021.599955 (PMC8354585; doi:10.3389/fimmu.2021.599955)
Supplement: Supplementary file 1 [file DataSheet_1.docx]

| **Patient 1** | **Before SLIT** | **1^st^ year of SLIT** | **2^nd^ year of SLIT** | **3^rd^ year of SLIT** | **Follow up 1^st^ year** | **Follow up 2^nd^  year** | **Follow up 3^rd^ year** |
| --- | --- | --- | --- | --- | --- | --- | --- |
| **Scale for assessing the mean adjusted symptom score with medication intake** | **26** | **16** | **6** | **6** | **2** | **4** | **2** |
| **ACT** | **22** | **25** | **25** | **25** | **25** | **25** | **25** |
| **VAS** | **10** | **7** | **2** | **μ3** | **1** | **1** | **1** |
| **CD4+ /μl** | **932** | **916** | **935** | **980** | **976** | **991** | **985** |
| **Viral load** | **negative** | **negative** | **negative** | **negative** | **negative** | **negative** | **negative** |

| **Patient 2** | **Before SLIT** | **1^st^ year of SLIT** | **2^nd^ year of SLIT** | **Follow up year** |
| --- | --- | --- | --- | --- |
| **Scale for assessing the mean adjusted symptom score with medication intake** | **25** | **12** | **3** | **4** |
| **VAS** | **9** | **5** | **1** | **1** |
| **CD4+/μl** | **519** | **623** | **631** | **674** |
| **Viral load** | **negative** | **negative** | **negative** | **negative** |

VAS - RHINOCONJUNCTIVITIS VISUAL AND ANALOGUE SCALE

ACT – ASTHMA CONTROL TEST

**SCALE FOR ASSESSING THE MEAN ADJUSTED SYMPTOM SCORE WITH MEDICATION INTAKE**

INSTRUCTIONS FOR ASSESSING RHINOCONJUNCTIVITIS SYMPTOMS

- Assess the following six symptoms of rhinoconjunctivitis observed during flowering period: sneezing, nasal discharge, itchy nose, nasal congestion, itchy eyes and watery eyes when they were most marked.
- Use the following quantitative indicators to assess each symptom:

1. = Absence of symptoms (no apparent signs/symptoms)
2. = Mild symptoms (sign/symptom is clearly present, but at a minimum; easily tolerated)
3. = Moderate symptoms (a clear sensation of signs/symptoms that are uncomfortable but tolerable)
4. = Marked symptoms (severely tolerated signs/symptoms; interfere with daily activities and (or) sleep)

INSTRUCTIONS FOR ASSESSING INTAKE OF RESCUE MEDICATIONS

- Use the following quantitative indicators to assess your treatment:

1. = Absence of additional therapy
2. = Antihistamines (AH)
3. = Local administration of glucocorticosteroids (lGCS)
4. = Oral administration of glucocorticosteroids (oGCS)

| **SCALE FOR ASSESSING THE MEAN ADJUSTED SYMPTOM SCORE WITH MEDICATION INTAKE** | | | | | | |
| --- | --- | --- | --- | --- | --- | --- |
| Sneezing | Nasal discharge | Itchy nose | Nasal congestion | Itchy eyes | Watery eyes | Rescue medications |
| 0 = Absence  1 = Mild  2 = Moderate  3 = Marked | 0 = Absence  1 = Mild  2 = Moderate  3 = Marked | 0 = Absence  1 = Mild  2 = Moderate  3 = Marked | 0 = Absence  1 = Mild  2 = Moderate  3 = Marked | 0 = Absence  1 = Mild  2 = Moderate  3 = Marked | 0 = Absence  1 = Mild  2 = Moderate  3 = Marked | 0 = Absence  2 = AH drugs  4 = lGCS  6 = oGCS  (Maximum: 6) |
|  |  |  |  |  |  |  |
| Score (Maximum: 24)  Максимум: 3 | | | | | | |
