# Supplementary material for: Case Report: Safe and Effective Sublingual Birch Allergen Immunotherapy in Two HIV-Positive Patients
Source: Front Immunol. 2021 Jul 27;12:599955. doi: 10.3389/fimmu.2021.599955 (PMC8354585; doi:10.3389/fimmu.2021.599955)
Supplement: Supplementary file 2 [file DataSheet_2.docx]

| Patient 1 | Before SLIT | 1st year of SLIT | 2nd year of SLIT | 3rd year of SLIT | Follow up 1st year | Follow up 2nd year | Follow up 3rd year |
| --- | --- | --- | --- | --- | --- | --- | --- |
| Scale for assessing the mean adjusted symptom score with medication intake | 26 | 16 | 6 | 6 | 2 | 4 | 2 |
| ACT | 22 | 25 | 25 | 25 | 25 | 25 | 25 |
| VAS | 10 | 7 | 2 | 3 | 1 | 1 | 1 |
| CD4+ /μl | 932 | 916 | 935 | 980 | 976 | 991 | 985 |
| Viral load | negative | negative | negative | negative | negative | negative | negative |

| Patient 2 | Before SLIT | 1st year of SLIT | 2nd year of SLIT | Follow up year |
| --- | --- | --- | --- | --- |
| Scale for assessing the mean adjusted symptom score with medication intake | 25 | 12 | 3 | 4 |
| VAS | 9 | 5 | 1 | 1 |
| CD4+/μl | 519 | 623 | 631 | 674 |
| Viral load | negative | negative | negative | negative |
